# Integration of Carbon Nanotubes in Microsystems: Local Growth and Electrical Properties of Contacts

**DOI:** 10.3390/ma6083094

**Published:** 2013-07-24

**Authors:** Tormod B. Haugen, Bao Q. Ta, Einar Halvorsen, Nils Hoivik, Knut E. Aasmundtveit

**Affiliations:** Department of Micro and Nano Systems Technology (IMST), Vestfold University College (HiVe), Raveien 197, 3184 Borre, P.O. Box 2243, Tønsberg N-3103, Norway; E-Mails: tormod.haugen@student.hive.no (T.B.H.); quoc.bao@hive.no (B.Q.T.); einar.halvorsen@hive.no (E.H.); nils.hoivik@hive.no (N.H.)

**Keywords:** carbon nanotubes, local synthesis, microheater, contacts, Schottky barrier

## Abstract

Carbon nanotubes (CNTs) have been directly grown onto a silicon microsystem by a local synthesis method. This method has potential for wafer-level complimentary metal-oxide-semiconductor (CMOS) transistor-compatible integration of CNTs into more complex Si microsystems; enabling, e.g., gas sensors at low cost. In this work, we demonstrate that the characteristics of CNTs grown on specific locations can be changed by tuning the synthesis conditions. We also investigate the role of the contact between CNTs and the Si microsystem; observing a large influence on the electrical characteristics of our devices. Different contact modes can render either an ohmic or Schottky-like rectifying characteristics.

## 1. Introduction

Ever since the discovery by Iijima in 1991 [1], carbon nanotubes (CNTs) have been subject to intensive research due to their unique properties. Their mechanical strength is remarkably high [2], and statements like “the strongest material known” and “a hundred times the strength of steel” are often seen. The thermal conductivity has been predicted to be significantly greater than that of diamond [3]. Maybe even more interesting are their unusual electronic properties. CNTs are one-dimensional conductors or quantum wires that can behave either as a metal or a semiconductor with a tunable band-gap.

CNTs have a great diversity of applications, including mechanical reinforcement in composites [4]; it can be used in field effect transistors [5] and as a smart material in micro- and nano-electromechanical systems (MEMS/NEMS) [6], to name just a few. Our paper addresses the latter example.

By integrating CNTs into microsystems, the nanostructures can effectively be connected to the macro world via a microscale platform. This opens the area for several applications. For example, the electrical conductivity of a CNT can be changed significantly both by adsorbed molecules and by straining the tubes [7,8]. Thus, ultra-high sensitivity chemical and mechanical sensors could be obtained by using CNTs as the sensing material. This paper presents parts of our work towards a long-term goal: to develop a low cost, CMOS/MEMS compatible, wafer level synthesis process for integrating CNTs on specific locations on a microsystem.

Successful CNT integration requires a synthesis method where we can control the type and orientation of the CNTs grown on specific locations on the microsystem. The local synthesis method, first presented by Englander* et al.* and Christensen* et al.* in 2003 [9,10], is a chemical vapor deposition (CVD) method for growing one-dimensional nanostructures. By heating specific locations on a microsystem only, the growth is confined to these locations, while the remaining areas remain at room temperature and will not experience any growth. Compared to synthesizing CNTs separately and assembling to a microsystem, this method of integration is considered to be a low cost process that easily can be scaled to the wafer level [11].

Contacts play a crucial role in electronic devices, and much work is published on the junctions formed when semiconducting single-walled CNTs contact metal electrodes [12,13,14]. Metal-semiconductor junctions result in rectifying Schottky barriers or ohmic I-V characteristics, depending on the work functions of the materials in contact. Schottky barriers have also been observed when multiwalled metallic CNTs are grown on semiconducting materials, such as silicon and silicon carbide, resulting in nonlinear I-V curves [15,16,17].

Previously, we have demonstrated local synthesis of CNTs on polycrystalline silicon microstructures [18]. In this paper, we present the results from CNTs grown locally on a microsystem and, specifically, microheaters fabricated from single crystalline silicon. Our focus is on how we can tune the local growth towards CNTs of particular shapes and diameters and what effects the contact between the CNT and the Si have on the electrical characteristics of our devices.

## 2. Experimental Section

**Figure 1:**
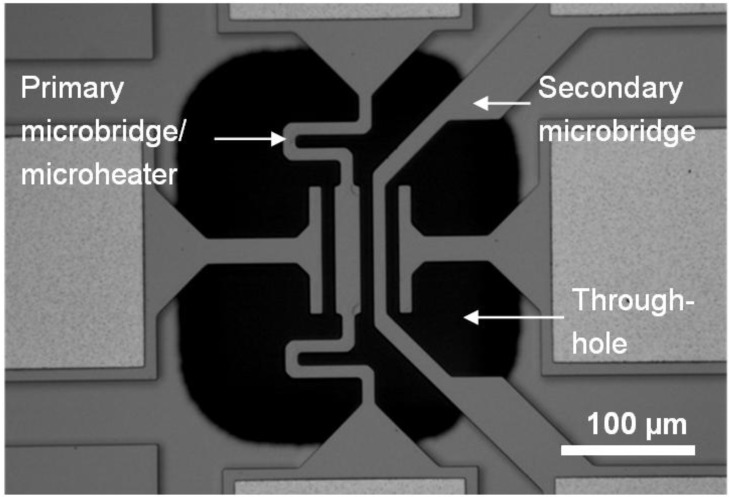
Optical micrograph of microsystem for carbon nanotube (CNT) growth, consisting of two suspended silicon microbridges. CNTs grow from the microheater (primary microbridge), guided by an electric field, and connect to the secondary microbridge. The through-hole in the Si substrate facilitates transmission microscopy.

We have designed different microsystems dedicated for CNT growth, which were fabricated on silicon-on-insulator (SOI) substrates with a commercial micromachining process [19]. Figure 1 shows one microsystem consisting of two suspended silicon microbridges, etched out from the 10 µm-thick single crystalline device layer. The device layer is heavily doped with phosphorous at the surface, resulting in a non-uniform doping profile through the layer. A 1 µm buried oxide layer electrically isolates the device layer from the handle wafer. A native oxide layer, 1–2 nm thick, is assumed to be present on the surface of the device layer.

The primary bridge serves as a microheater from which we want the CNTs to grow. By passing a current through the suspended bridge, the center easily reaches a temperature suitable for CNT growth, while the bulk of the chip remains at room temperature. The secondary bridge is separated from the first bridge by a gap of 10 µm. Ideally, the CNTs grow from the first bridge, across the gap and connect to the secondary bridge. The microsystem also includes two electrodes on each side of the microbridges, as seen in Figure 1, but these were not used in this work.

The microbridges are suspended above a hole through the entire wafer, making transmission imaging of the CNTs possible. The microheater is made spring-like to absorb thermal expansions during operation. By increasing the width of the microheater from 10 µm at the ends to 20 µm at the center, the current density in this region will be lower, and thus, less heat is generated. Therefore, our microheaters obtain a much more uniform temperature profile than those with a constant width, and we expect uniform growth over a well-defined region. Finite element method (FEM) simulations with the software COMSOL Multiphysics were used in the design phase to estimate the temperature profile along the microheater and to ensure that the highest temperature remained in the center region.

A thin (1–3 nm) layer of Fe, to serve as the catalyst material for CNT growth, is thermally evaporated on the microsystems (Moorfield Associates Minilab T25M). No patterning of the Fe layer is needed, as nanoparticles are formed on the microheater when the thin film is heated. A chip containing 20 microsystems is then mounted on a ceramic chip carrier, and electrical connections from the chip to the carrier are done by wire bonding (F&K Delvotec 5610).

CNT synthesis is done with a method similar to that we have presented before [18]. The sample is placed in a room-temperature reaction chamber (~200 cm^3^), which is purged for a few seconds, before argon is introduced at a flow rate of 50 ccm and a pressure of 0.4 bar. The primary bridge is gradually heated to around 900 °C at a rate of approximately 5 °C/s, by increasing the current through it. Acetylene (C_2_H_2_) gas, the precursor gas for CNT growth, is then immediately introduced to the chamber at a flow rate of 50 ccm. The overall pressure is still kept at 0.4 bar.

A DC voltage (1–10 V) is applied between the primary and secondary microbridge, to create an electric field in the gap between the bridges. The field is intended to guide the CNT growth from the primary to the secondary bridge. The voltage difference between the primary and secondary bridge also serves as a method for monitoring the number of CNT connections established during synthesis. By measuring the current between the two bridges during the synthesis process, any CNT connection is detected as a jump in the current.

The temperature of the microheater during synthesis is determined by measuring the resistance of the microheater. The resistivity of silicon, and of any semiconductor in general, will greatly vary with temperature. Thus, by comparing the measured resistance with results from an FEM model that includes both temperature-dependent charge carrier concentrations and mobilities, we can determine the synthesis temperature with a simple setup. Further details of this method are described in [20].

The local CNT growth was inspected with Scanning Electron Microscope (SEM, Philips XL30) and S(T)EM (Hitachi S5500). No preparation of the samples was required prior to transmission mode imaging, as the CNTs were grown above a through-hole in the wafer.

Current-voltage (I-V) measurements were made between the two microbridges to investigate the electrical characteristics of the Si/CNT/Si systems. I-V data were obtained at room temperature using a Keithley 2602 source meter.

## 3. Results and Discussion

### 3.1. Types of CNTs

**Figure 2:**
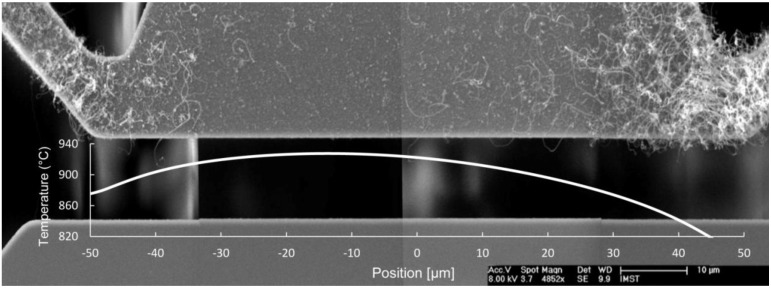
Scanning Electron Microscope (SEM) micrograph of microheater after CNT growth with simulated temperature profile, showing the asymmetrical temperature distribution, due to the Thomson effect.

Figure 2 shows an SEM image of a microheater with CNT growth on its surface. The asymmetric growth along the heater is explained by the Thomson effect, a thermoelectric effect that is observed when an electric current is passed through a conductor along which there also is a temperature gradient. The result is that the hottest point is shifted from the center of the bridge by a distance known as the Thomson shift [21].

Figure 2 also shows the temperature profile along the microheater, obtained by FEM simulations in which the Thomson effect was included. The asymmetry in the temperature profile corresponds well to the asymmetry in the growth. The highest temperature in the simulated profile is shifted 15 µm left of the center of the microbridge, and at this position, the lowest CNT growth density is observed. Still, the growth seems to be quite uniform in the region from −35 to +10 µm, where the temperature is in the range of 910 to 930 °C.

**Figure 3:**
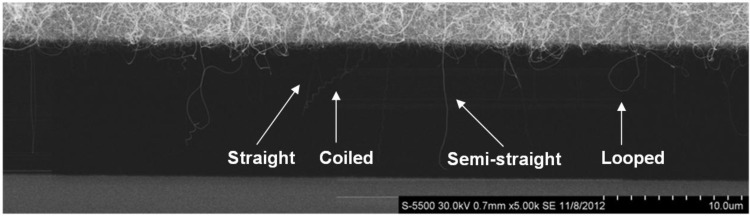
Collection of several SEM images showing CNTs of different shapes growing from the microheater (top) and across the gap towards the secondary microbridge (bottom). Synthesis temperature is 770 °C.

Other microheaters we used for CNT growth, with slightly different designs, had regions of uniform growth over a larger part of the microheater, up to 100 µm. Figure 3 shows a collection of several SEM images along a microheater that was subject to CNT synthesis at 770 °C. CNTs of different shapes can be seen to grow across the gap and, in some cases, also connect to the secondary microbridge.

**Figure 4:**
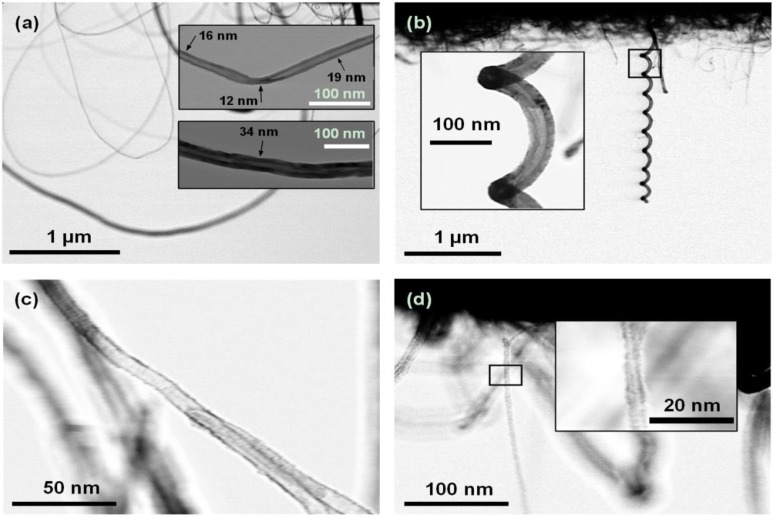
Transmission mode SEM images of different types of suspended CNTs. (**a**) looped; (**b**) coiled; (**c**) semi-straight; and (**d**) straight CNTs.

A more detailed study with respect to the structure and defects of the tubes and the covering of amorphous carbon is presented elsewhere [22,23]. Here, we have categorized the suspended CNTs into four groups based on their fundamental shape: (1) looped; (2) coiled; (3) semi-straight; and (4) straight. Examples of CNTs in the different categories are shown in the SEM images in Figure 4, and structural parameters are summarized in Table 1.

**Table 1:** Classifications of CNTs in groups based on their fundamental shape.

CNT Shape	Looped	Coiled	Semi-straight	Straight
Observed diameter range [nm]	12–60	25–100	6–30	1–5
Apparent wall thickness * [nm]	3–15	6–14	1–6	1
Relative amount at 770 °C	57%	7%	33%	3%

* The darker region on each side of the tube as observed in SEM transmission imaging is here defined as “apparent wall thickness”.

Looped tubes are recognized as first growing outwards from the microheater, typically 1 to 3 µm, and, then, make a turn and grow back. From the top inset in Figure 4a, a local decrease in the tube diameter can be observed at the point where the growth direction changes. The tube diameter is 15 nm and 19 nm on either side of the kink, but is reduced to 12 nm at the kink. This was also observed in other tubes, where there was a sudden change in the growth direction occurred. Changes in the growth direction and diameter are accomplished by defects in the hexagonal carbon lattice, and at the kinks, we thus expect a high density of defects in the CNT material.

Helically coiled CNTs were seen to grow with pitches ranging from a few hundred nanometers to more than one micrometer and did in some cases connect the two microbridges. Coiled CNTs can show a remarkable regularity, as shown in Figure 4b. Tubes were classified as semi-straight when they made no loops or coils, but their growth direction deviated from a straight line. Straight tubes were most difficult to resolve, due to their small diameters.

Most of the CNTs are believed to be multiwalled, based upon the measured diameters. The apparent wall thickness seen in the transmission-mode S(T)EM images also indicate a high number of walls, taking into account the interlayer spacing of 3.4 Å often measured on multi-walled tubes [24]. However, it is not possible to discern from the images if the tubes actually consist of multiple walls or just one thick wall with a high degree of disorder. Single-walled nanotubes have been reported to have diameters in the range 0.7–10 nm, and in most cases, less than 2 nm [25], so there is a possibility that the smallest CNTs we have observed are single-walled. High-resolution Transmission Electron Microscope (TEM) imaging would therefore be a desired tool for future work, to learn more about the structure of the tubes.

### 3.2. Effects of Synthesis Temperature on CNT Shape, Diameter and Growth Densities.

**Figure 5:**
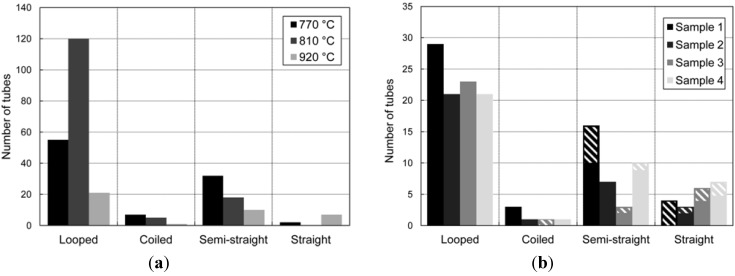
Number of CNTs, in different categories, growing in the gap between the primary and secondary bridge. (**a**) Samples grown at different temperatures; (**b**) samples grown at similar temperatures. In (**b**), the hatched regions represent the number of tubes that connected to the secondary bridge.

The number of CNTs growing in the gap from the primary silicon bridge and towards the secondary bridge was counted for three samples grown at different temperatures, but with all other synthesis parameters kept similar. The distribution of tubes in the categories described in the previous section is shown in Figure 5a.

The ratio between straight and looped tubes is largest for the sample grown at the highest temperature. We find this to be of particular interest in the sense that the looped tubes represent the highest degree of disorder, and in very few cases, they connect to the secondary bridge and, thus, can be regarded as unwanted. The straight tubes, on the other hand, did, to a much larger extent, connect to the secondary bridge and are favorable from an application point of view.

The increase in the count of looped tubes grown at temperatures between 770 and 810 °C can be explained by the fact that the length of the tubes increased; thus, more tubes grew from the top surface of the microheater into the gap, rather than that the increase in temperature resulted in more tubes in total.

The growth repeatability was investigated by collecting the same data from four different samples grown under identical synthesis conditions, all with a microheater peak temperature of 920 °C. The results are shown in Figure 5b. An interesting parameter for future applications is the growth density of straight tubes that grow across the 10 µm gap and connect to the secondary structure. For the four samples in Figure 4b, the average density is 5.6 tubes/100 µm. Dittmer* et al.* have observed a much higher growth density of CNTs between similar microstructures to ours, but their gap was only 3 µm [26].

It should be noted that for all samples, there was a temperature gradient along the microheater, as was shown in Figure 2, and that the temperatures given in Figure 5 are the peak temperatures. For the samples in Figure 5 with a peak temperature of 920 °C, the looped tubes were mostly found at the two ends of the microheater, where the temperatures were lower, whereas the straight and semi-straight tubes grew closer to the center of the bridge.

The change in shape of the suspended CNTs from looped towards straight with increasing synthesis temperature can be explained from the diameter of the CNTs and the effect of alignment with the electric field during growth. Higher synthesis temperatures resulted in tubes with smaller diameters, which is in agreement with other work [27]. Ongoing research shows that the CNT diameter has a big influence on the alignment effect, and there seems to be a critical diameter above which the CNTs are no longer guided by the electric field [28]. From the results presented above, the looped tubes, showing no alignment to the applied E-field, had diameters in the range 12–60 nm. Straight tubes, aligned with the E-field, in general, all had a diameter less than 5 nm.

For the CNTs growing on the heater, no favored growth orientation could be observed, and the tubes seemed to stick together in bundles (Figure 2), which also has been observed by others [26,29]. It can be explained by van der Waals interactions between tubes and the surface of the microheater and also between adjacent CNTs, holding the nanotubes in place and preventing alignment with the electric field.

**Figure 6:**
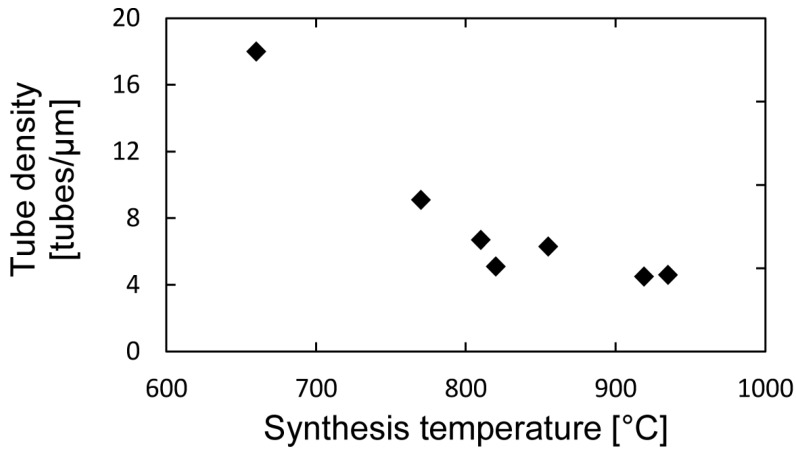
Growth density of randomly oriented CNTs on the microheater surface as a function of synthesis temperature.

From Figure 2, it can also be seen that the growth density of looped tubes on the surface of the microheater decreases with temperature. This was further investigated on a selected number of samples grown at different temperatures, by counting the number of tubes growing across a 5 µm line at the center of the microheaters. The results are shown in Figure 6 and support the idea of reducing the number of unwanted, randomly-oriented CNTs by increasing the synthesis temperature.

We also observed longer tubes on the microheater surface where the temperature is increased in the range of 660 to 885 °C. This trend is in agreement with results from Engstrøm* et al.* [30], who found that for CNTs growing vertically on a microheater, the tube length was increasing almost linearly with temperature from 575 to 800°C. Above this temperature, they report that only a thin film of randomly oriented CNTs grew. This is similar to the observations of reduced growth density at higher temperatures in our work; however, the effect is not as abrupt in our experiment as they found.

### 3.3. CNT/Si Contacts

**Figure 7:**
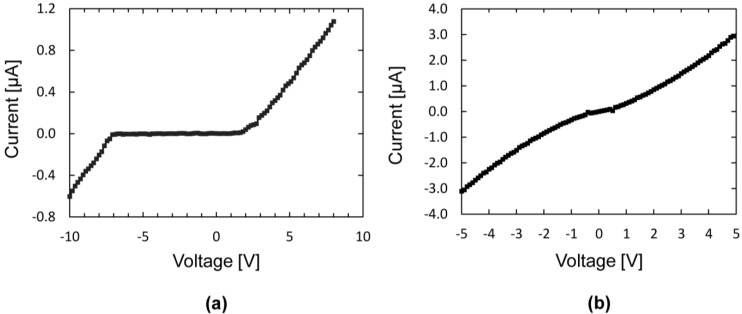
Measured current-voltage relationship for two Si/CNT/Si systems. (**a**) Rectifying characteristics, having a conducting region for voltages less than −7 V and greater than +2 V; (**b**) Near-ohmic characteristics with deviations from a linear behavior similar to (**a**).

Figure 7 shows the results from I-V measurements on two different Si/CNT/Si systems grown under similar conditions. The characteristics in Figure 7a resembles rectifying contacts similar to two diodes connected back to back, each entering the conducting state in the reverse direction at bias voltages of 7 and 2 V. The curve in Figure 7b has a near-ohmic I-V characteristics, but also, this curve shows deviations from a linear behavior similar to the curve in Figure 7a.

**Figure 8:**
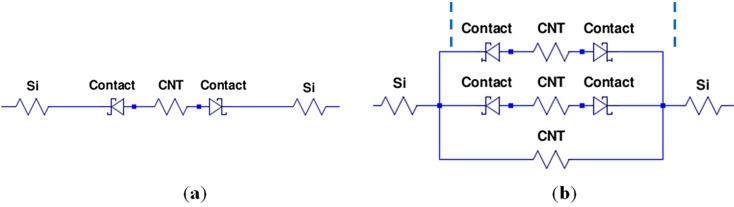
Electrical model representing the Si/CNT/Si system yielding (**a**) rectifying characteristics; and (**b**) near-ohmic characteristics.

An electrical model equivalent to the rectifying Si/CNT/Si system is presented in Figure 8a. The Si/CNT contacts are represented by two Schottky diodes connected back to back. For each diode, the reverse breakdown voltage, *V*_br_, can be found from the voltage at the kinks in the I-V curves. Our samples with rectifying characteristics had reverse breakdown voltages in the range of 2 to 12 V.

Resistors represent the resistance of the Si microbridges, the CNT resistance and the two contact resistances. The total resistance was extracted from the I-V curves by taking the reciprocal of the slope of the I-V curve in the conducting region,* i.e.*, at |*V*| > *V*_br_, and had values in the range of 500 to 2.5 MΩ. The Si microbridges have resistances of a few hundred ohms and are, thus, negligible compared to the total resistance. However, the CNT resistance and the Si-CNT contact resistance cannot be separated in these measurements.

Figure 8b shows an electrical model representing the near-ohmic systems. A single resistor represents a perfect ohmic contact. Adding multiple Schottky diodes and resistors in parallel, with slightly different breakdown voltages and resistances, change the shape of the I-V curve towards the near-ohmic case. Physically, this represents several CNTs connecting the two microbridges, some having rectifying contacts and one having pure ohmic contacts.

**Figure 9:**
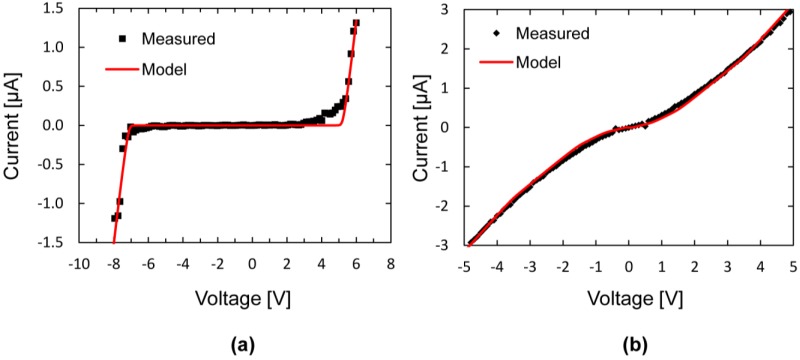
I-V curves from the electrical model compared to measured values. (**a**) Rectifying and (**b**) near-ohmic characteristics.

I-V curves were obtained from the electrical models with a circuit simulation software (LTspice IV). Figure 9a shows the characteristics of the model in Figure 8a with Schottky diode breakdown voltages of 6 and 7 V and a total resistance of 500 kΩ. Figure 9b shows the characteristics of the model in Figure 8b. Both curves match the experimental data well.

**Figure 10:**
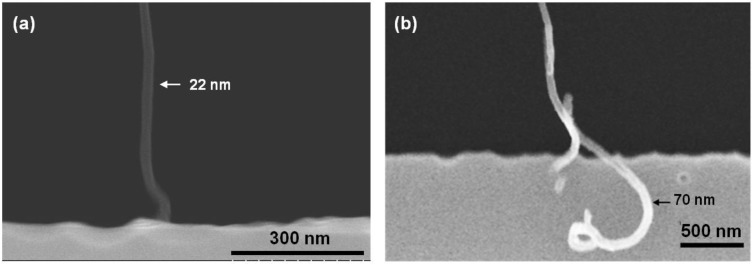
SEM pictures of two observed contact modes for CNTs connecting to the secondary bridge. (**a**) CNT in end contact with the sidewall of Si microbridge; (**b**) CNT in side contact with the top surface of the Si microbridge.

The difference seen in I-V characteristics can be explained from the contact mode between the CNTs and the silicon microbridges. As depicted in Figure 10, the contact can be made both at the sidewall and the top surface, and it is the non-uniformity in the doping concentration along the thickness direction of the microbridges that explains the different contacts. The doping concentration at the surface is on the order of 10^2^^0^ cm^−3^ [19] and decreases with distance down into the microbridges.

When metallic CNTs contact the n-type silicon microbridges, a Schottky barrier of potential height, $\phi_{B}$, is formed at each contact, retarding the electron flow from the CNT into silicon, but allowing electron flow the opposite way. In the ideal case, when Fermi-level pinning from interface states and barrier lowering from image charges are neglected [31], the barrier height, $\phi_{B}=\phi_{CNT}-\chi$. *q*$\phi_{CNT}$, is the CNT work function for which we can use the value of graphite (4.4 eV [32]) and  qχ is the electron affinity of silicon (4.05 eV [33]). For either polarities of the applied voltage, one barrier will always be reverse biased, and thus, no significant current should run in the circuit. However, if the reverse bias over a Schottky diode is high enough, breakdown occurs, and the diode is conducting in the reverse direction.

Reverse breakdown voltages for CNT/Si contacts were estimated with an avalanche breakdown model [34], which gives values in the range of 4 to 15 V for moderate doping concentrations of 10^16^ to 10^18^ cm^−3^, similar to the doping of a short distance from the surface of the microbridges. This result is consistent with the breakdown voltages we observe from the I-V measurements.

If the CNTs, however, contact to regions of silicon that are heavily doped, the contacts can be ohmic or near-ohmic. This happens because the barrier width is greatly reduced, and electrons can tunnel straight through the thin barrier [31].

Based on these results, it is reasonable to assume that it is the variations in the doping concentration that renders the different contacts and that systems with many CNT connections, therefore, can have a great variety of I-V characteristics. It is difficult to control the contact position between the CNT and the silicon with respect to the doping concentration gradient. In future applications, we therefore want to use microsystems with a more uniform, well-defined doping level to have control of the electrical characteristics.

In our previous work, we have grown CNTs between polycrystalline microbridges, 2 µm-thick with uniformly high doping concentration [18]. Here, all I-V measurements showed near-ohmic characteristics, strengthening our theory that the contacts are rectifying when formed at regions with lower doping concentrations. Note that for all these samples, the deviations from true ohmic (linear) behavior is an S-shaped modulation of the IV-curve, qualitatively similar to the weakly rectifying behavior, as shown in Figure 7b. This shows that also at high Si doping concentrations, a remnant of the effect of Schottky junctions can be observed.

The effect of interfacial layers on the contact, like the native oxide on the silicon surface and amorphous carbon covering the tubes, has so far been neglected in our discussion, as we have assumed electrons tunnel straight through these thin layers. At this stage, we have no information on the amount of amorphous carbon, how much oxide is present on either Si-side after synthesis or if some CNTs penetrate the oxide for better contact. However, it is likely that there is a 1–2 nm oxide layer present between the CNT and the Si and that the contact must be treated as a metal-insulator-semiconductor (MIS) tunneling diode. Compared to conventional metal-semiconductor (MS) diodes, MIS diodes have reduced current, because of the interfacial layer, lower barrier height and higher ideality factor [34]. Tunneling through the oxide will contribute to a high series resistance, due to the small contact area [15], and can explain the high resistance we observed in our measurements.

## 4. Conclusions

Local synthesis at different temperatures resulted in CNTs of various shapes growing along the silicon growth structure, with regions of uniform growth up to 100 µm. Tubes were classified as looped, coiled, semi-straight and straight, based on their fundamental shape. The growth density of unwanted looped tubes was found to decrease with synthesis temperature, whereas the relative amount of straight tubes increased with temperature.

Electrical characterization showed that the Si/CNT contact is rectifying and that the total Si/CNT/Si system can be modeled as two Schottky diodes connected back to back and in series with an equivalent resistor. The calculated doping dependence of the reverse breakdown voltage of the Schottky diodes was found to match the measured results. I-V characteristics of the CNT/Si contact changed from near-ohmic towards rectifying when the contact was formed at positions where the silicon had a lower doping concentration. The presence and effects of interfacial layers at the contacts are currently not fully known and are part of our future work.

The information obtained from this work allows a better prediction of the results from CNT growth with given parameters in our local synthesis/direct integration approach. As such, it is an important contribution towards our long-term goal of a reliable and predictable, low-cost process for CNT integration in Si microsystems.
